# A Comprehensive Review on Significance and Advancements of Antimicrobial Agents in Biodegradable Food Packaging

**DOI:** 10.3390/antibiotics12060968

**Published:** 2023-05-26

**Authors:** Ipsheta Bose, Swarup Roy, Vinay Kumar Pandey, Rahul Singh

**Affiliations:** 1 School of Bioengineering and Food Technology, Shoolini University, Solan 173229, India; ipsheta18@gmail.com; 2 Department of Food Technology and Nutrition, School of Agriculture, Lovely Professional University, Phagwara 144411, India; 3 Department of Bioengineering, Integral University, Lucknow 226026, India; vinaypandey794@gmail.com; 4 Department of Biotechnology, Axis Institute of Higher Education, Kanpur 209402, India

**Keywords:** biodegradable, biopolymers, antimicrobial agent, active packaging, food industry

## Abstract

Food waste is key global problem and more than 90% of the leftover waste produced by food packaging factories is dumped in landfills. Foods packaged using eco-friendly materials have a longer shelf life as a result of the increased need for high-quality and secure packaging materials. For packaging purposes, natural foundation materials are required, as well as active substances that can prolong the freshness of the food items. Antimicrobial packaging is one such advancement in the area of active packaging. Biodegradable packaging is a basic form of packaging that will naturally degrade and disintegrate in due course of time. A developing trend in the active and smart food packaging sector is the use of natural antioxidant chemicals and inorganic nanoparticles (NPs). The potential for active food packaging applications has been highlighted by the incorporation of these materials, such as polysaccharides and proteins, in biobased and degradable matrices, because of their stronger antibacterial and antioxidant properties, UV-light obstruction, water vapor permeability, oxygen scavenging, and low environmental impact. The present review highlights the use of antimicrobial agents and nanoparticles in food packaging, which helps to prevent undesirable changes in the food, such as off flavors, colour changes, or the occurrence of any foodborne outcomes. This review attempts to cover the most recent advancements in antimicrobial packaging, whether edible or not, employing both conventional and novel polymers as support, with a focus on natural and biodegradable ingredients.

## 1. Introduction

Food packaging is described as enclosing meals to defend them from tampering or infection from physical, chemical, and biological sources, where active packaging is considered the most desirable packaging system used for retaining meals products [1]. The four essential purposes of traditional food packaging are containment and communication, protection and preservation, convenience and marketing [2]. Food packaging has to meet some criteria, including legislation, protection, and plenty of different situations, since it is required to be innovative, clean to apply, and have an appealing design. One of the principal duties of packaging within the food enterprise is to guard the product from chemical, mechanical, and microbiological impact, and additionally maintain the freshness of the product and its dietary value. Good packaging prevents waste and guarantees that the meals keep their flavor until some stage in their shelf life. Despite its significance and the important function that packaging plays, it is often seen as, at best, incredibly superfluous, and, at worst, a critical waste of resources and an environmental menace. The world loses an astounding quantity of food every year. Globally, around USD 750 billion worth of food is frittered away each year throughout the entire supply chain, among which up to 25% of residential meal waste is because of packaging size or design, for example, meals spoiling because of loss of packaging, condiments sticking to the perimeter and bottom of packing containers, or the inability to pack bulk clean ingredients for well-timed consumption [3,4]. Packaging waste, that from non-biodegradable polymers in particular, has ended up generating a large part of municipal solid waste, which results in a number of growing environmental concerns.

A significant waste management difficulty is presented by the very evident source of litter composed of leftover packaging. The petroleum-based polymer polyethylene (PE), which is one of these materials, is most frequently utilized in packaging applications [5]. After being dumped on land or along the coast, petroleum-based polymers such as PE are incredibly resistant to biodegradation, resulting in various degrees of contamination. To cope with this problem, a whole lot of interest has been paid to developing biodegradable polymers from renewable resources in recent years [6]. Numerous studies have shown great possibilities of new food packaging technologies using biodegradable and bio-based packaging material [7]. To lessen the environmental impact and petro-dependence, non-biodegradable plastics can be replaced with biopolymer. Biopolymers are the most promising alternative to synthetic plastic material for packaging that fully decomposes or can be composted [8]. Similar to this, there is current interest in the development of novel and more complex methods to prevent food from becoming contaminated by pathogenic microorganisms as a result of antimicrobial packaging, which refers to packaging systems that can prevent or eliminate pathogenic microorganisms from existing in the food [7]. However, the demand for environmentally friendly, affordable, sustainable materials has given a variety of naturally occurring antimicrobial compounds a fresh, intriguing outlook.

The growing trend also brings us to a point where we need to focus on antimicrobial packaging because spoilage due to microbial growth is one of the major problems faced by the food industry. These types of microbial growth elevate the risk of food-borne diseases and also alter the nutritional properties of the food product. In this sense, applying natural antimicrobial compounds to the packaging material will create a better packaging technology. As we all know that food packaging innovations have gradually accelerated towards the development of intelligent packaging throughout the beginning of the current millennium, the general motive of this paper is to provide an overview of ongoing research and the latest technologies which show the perspective of the next generation of intelligent, active food packaging systems that will sense various changes in the packaging or its environment [7]. An important development trend for the evolution of packaging is the incorporation of extremely effective antibacterial nanoparticles, antifungals, and antioxidants to biodegradable and environmentally friendly green polymers [2]. Due to increased awareness and needs for sustainable active packaging that may maintain the quality and extend the shelf life of foods and products, the development of antimicrobial packaging has been advancing quickly. Antimicrobial agents, if infused into the packing film, will prevent or eradicate the pathogenic microorganisms that cause food spoilage and sickness [3]. The uses of this novel packaging method for food, however, are still few. Even if this idea gains popularity and appears promising on paper, it is difficult to manage how much of an active ingredient is released into food, and little research has attempted to address this issue [2]. One effective way for expanding the shelf life of packaged food goods is the inclusion of active ingredients into natural and synthetic polymers and the development of coatings/films [6]. Theoretically, antimicrobial drugs ought to be given out at a controlled rate. Additionally, to prevent negative effects on sensory and toxicological qualities, the concentration of the released antimicrobial agent should be neither too high nor too low [3]. The use of cutting-edge active packaging methods in conjunction with innovative antimicrobial drugs has gained popularity. Sorbic acid is known to prevent the germination of bacterial spores, and both fungal and bacterial cell development is inhibited by organic acids. The fundamental cause of the inhibitory effect of organic acids is the compound’s entry into the plasma membrane in its protonated form. As a result, the acid will dissociate when it comes into contact with the higher pH inside the cell, releasing charged protons and anions that cannot flow through the plasma membrane. Additionally, the inhibiting effect of organic acids on yeasts may be brought on by the start of a stressful reaction that is so demanding that attempts to restore equilibrium and avoid other negative effects result in the depletion of resources [8].

By utilizing the various antimicrobial packaging solutions, sustainable active packaging that meets industry standards for higher safety and quality as well as a longer shelf life will be even better. By using renewable and biodegradable polymers rather than typical synthetic ones, antimicrobial packaging not only has a number of benefits but also protects the environment by lowering the amount of plastic pollution produced by humans. The World Wildlife Fund also noted in 2018 that almost 60% of the estimated 8 million tonnes of plastic that enter the oceans annually came from China, Indonesia, Malaysia, the Philippines, Thailand, and Vietnam [9]. The considerable amount of extremely hazardous emissions, composting management concerns, and changes in the carbon dioxide cycle are mostly to blame for this environmental threat. Therefore, this phenomenon has drawn the attention of numerous researchers who are working to produce sustainable, active packaging materials. As a result, packaging design should take user-friendliness and environmental sustainability into account in addition to shelf life, cost, and protection [10].

One effective method for extending the shelf life of packaged food goods is the inclusion of active chemicals into natural and synthetic polymers and the development of film or coatings. A variety of polymers, including agar, pullulan, carrageenan, alginate, cellulose acetate soy protein, and chitosan, have been implemented to create films that contain antibacterial ingredients [6]. The advantage of these biopolymers is their sustainability, renewability as well as biodegradability, which make them superior over the synthetic petrochemical-derived plastics [2]. Recent reports suggest the bio-based polymers or bioplastics are easily degradable in soil and sea water, although the rate of degradation is highly dependent on the type of polymers and the other fillers present in the composite [8]. Most of the bio-based polymers are highly biodegradable and their use in the packaging is not harmful to our environment nor for our health. In order to allow the integrated antimicrobial peptide to diffuse and protect against bacteria that may be present on the food surface, appropriate contact between the active packing material and the food product must be ensured [3]. 

Antimicrobial peptides are primarily incorporated into packaging materials using three different techniques: direct integration into polymer matrix, immobilization, and coating on the material’s surface [11]. To ensure the proper protective function during the anticipated shelf life, a balance between the development of microbial kinetics and the controlled release rate should be created. Controlling the rate at which antimicrobial agents leak from packaging and then diffuse into food products is thus one of the most intriguing difficulties in the practical application of antimicrobial systems. The effectiveness of active packaging systems can be impacted by such polymer-specific characteristics as mass transport, permeability, sorption, and migration. Traditional polymers can be thermally combined with tiny antibacterial agents. In this instance, the polymeric material’s amorphous zones could accommodate the antimicrobial chemicals without appreciably affecting the polymer’s internal structure [12]. A rising number of people are interested in developing and using controlled-release strategies for such compounds by making slight adjustments to the chemical and physical characteristics of the hosting materials to make them meet the specifications for food packaging materials [13]. 

The summary of current developments and uses of antimicrobial biodegradable films in the packaging sector, as well as the development of nanotechnology to produce highly effective new, bio-based packaging solutions, are the main topics of this review. Because of this, the impact of appealing product packaging on consumer purchase behaviour is significant. The majority of consumers prefer the new, added value that modern packaging technology has over conventional packaging. To meet the rising demand for packaged, ready-to-eat foods, which is regarded as a major driver of future packaging trends, the integration of these responsive technologies into food packaging will have a significant impact on the food processing sectors.

In general, antimicrobial agents can inhibit microbial growth in a variety of ways, including by changing the structure of proteins through modification or denaturation; altering the proteins or lipids in cell membranes; preventing the synthesis of components of cell walls; hindering the replication, transcription, and translation of nucleic acids; and interfering with cellular metabolism. Particularly in the context of perishable foods, the antimicrobial agents included into packing materials greatly increase microbiological safety, shelf life, and quality [14]. Antimicrobial agents can change the structure and engineering characteristics of packaging materials, including their tensile strength, gas permeability, and optical, thermal, morphological, and physical properties [15]. Clarke et al. 2016, investigated the physical characteristics of produced gelatin-based films containing the antibacterial agents Articoat DLP 02 (AR), Artemix Consa 152/NL (AXE), Auranta FV (AFV), and sodium octanoate (SO) [16]. The prepared films reported values for thickness, color, and transparency that were significantly greater than those of the control films. The very first polymeric nanocomposite to hit the market was clay, a revolutionary material for food packaging, with MMT being the most popular type. According to reports, the nanoclay contained in food packaging films reduces the rate of gas transmission to maintain the freshness and extend the shelf life of foods that are susceptible to oxygen [17]. Strong antibacterial activity against foodborne pathogenic microorganisms was demonstrated using gelatin-based nanocomposite films mixed with specific organic fillers and nanometals such titanium dioxide (TiO_2_), nanocopper (CuNPs), nanosilver (AgNPs), and zinc oxide nanoparticles (ZnO NPs). The active food packaging sector has a high potential for using the antibacterial gelatin-based nanocomposite films [18].

Innovative antimicrobial food packaging films are often made of bio-based polymers, which are biocompatible and safe for human consumption. The majority of them are edible and generally recognized as safe (GRAS)-classified. For instance, chitosan and cellulose are GRAS on the micron scale. Although nanocellulose shows little cytotoxicity, it may nevertheless affect the population of gut bacteria and change intestinal function by impairing nutritional absorption [19]. However, in addition to its benefits, nanotechnology also poses certain threats and has unfavorable effects on both the environment and people. The non-degradable and persistent character of nanomaterials is primarily responsible for their toxicological effects, whereas the advantageous elements of the unique properties of nanoparticles are provided by their tiny size and high surface area. However, it also has negative side effects, such as a high reactivity when interacting with biological elements [20]. Due to their high level of activity, nanoparticles can easily bypass blood arteries and membrane barriers, which could have a variety of hazardous effects. Additionally, given their small size and huge surface area, nanoparticles have unique biokinetic properties that may promote their migration from packing materials to food products as well as their likelihood of free movement and cell penetration in the body [21]. The toxicological concern of Ag, ZnO, and CuO nanoparticles is among the most studied and researched, as various studies indicate a strong relation between decreasing nanoparticle size and increasing phytotoxicity [22]. The toxicity of nanoparticles has been found to be inversely related to particle size, meaning that toxicity rises as particle size decreases. For instance, compared to silver nanoparticles with a diameter of 100 nm, 20 nm silver nanoparticles are more hazardous to lung tissue. Therefore, the transfer of nano-components from packaging to food may result in negative health effects. A few reports have also indicated that these components may be genotoxic and carcinogenic [23]. Studies on essential oils show that at the concentrations used in food packaging, they are not hazardous. There are some worries that when essential oils are used to prevent pathogenic bacteria, they may also have a harmful effect on beneficial microorganisms [24]. Last but not least, before nanomaterials are actually commercialized, more research on the health and environmental safety of these materials is still required.

Numerous review articles on the use of antimicrobial compounds in packaging materials have recently been published that mainly highlight how well the substance reduces food spoilage and discuss how to include or use these ingredients in packaging materials. However, new developments in nanotechnology and the creation of biodegradable packaging material using different compounds have gained the limelight. In this review article, we briefly discuss various organic and chemical-based antimicrobial compounds which can be utilized in forming biodegradable packaging material, while highlighting recent research studies on the application of nanotechnology to build novel bio-based packaging solutions; this study assesses the current situation and applications of antimicrobial biodegradable films in the food packaging business.

## 2. Classification of Antimicrobial Agents

The main cause of food rotting is microbial infection. Food-borne microbial illnesses are treated using antimicrobial medicines. They are also utilized in the food packaging sector to create antimicrobial packaging films that protect food’s structure, texture, color, and nutritional content. Food demand is increasing in tandem with population growth. Food waste must be stopped, and rotting must be avoided. The majority of the food spoils while being harvested, transported, and distributed. It is important to solve this significant issue. The most convenient technique to lessen food degradation and contamination is to add antibacterial agents. Each antimicrobial agent has a distinct mechanism and responds to various kinds of microbes in a different way. In this instance, the sorts of antimicrobials might sometimes place limitations on how they can be used. A suitably continuous, sticky, and cohesive matrix must be formed by at least one component during the production of antimicrobial films. A formulation like this includes a plasticizer (sorbitol, glycerol, and water), a pH-adjusting agent (acid, sodium hydroxide, and others), a film-forming agent (polymer), a solvent (ethanol, water, and others), and an antibacterial agent [11]. Depending on what the packaging material is being used for, different antimicrobial agents may be chosen. One of the most demanding methods for food preservation, food packaging, has undergone several alterations to support the characteristics and qualities of antibacterial materials. Due to historical reasons, low cost, and effective barrier properties, petrochemical polymers are currently the basis for the majority of food packaging materials. These polymers are not biodegradable, and they have already caused significant environmental problems in terms of short- and long-term contamination [12]. Antimicrobial packaging containing antimicrobial agents interacts with packed food in order to take effect, such as to inhibit, reduce, or retard microbial growth along with increasing shelf life of the food product [13]. Food-borne microbial diseases are treated with antimicrobial agents. They also benefit the food packaging sector, as they are utilized for the manufacture of antimicrobial packaging films which protect the structure, texture, color, and nutrient value of food. The antimicrobial packaging techniques fall into two categories. The first type is represented by packaging materials that allow for direct contact between the preserved food and an antimicrobial surface, allowing for the migration of active ingredients into the food. These containers are used for food that has been vacuum-sealed or wrapped in foil. The modified atmospheric packaging (MAP) is a second tactic that places the antimicrobial agent inside the package but out of direct contact with the food [14,15]. Bioactive agents can be added directly to packing compounds to create antimicrobial packaging, they can be coated onto packaging surfaces to create antimicrobial packaging, or they can be incorporated into films made of antimicrobial polymers [16]. Organic acids, enzymes, bacteriocins, fungicides, natural extracts, ions, ethanol, polyphenols, protein hydrolysates, and other substances can all be used as active agents [17,18].

In a recent research work it has been highlighted that there are a few salts and organic acids which possess strong antimicrobial properties; some agents include sorbic, benzoic, acetic, propionic, and ascorbic acids, and they can change the transport and permeability of membranes as well as the pH levels inside cells [19]. Essential oils and aqueous or alcoholic extracts from herbs, spices, and plants such as basil, eucalyptus, thyme, mustard, and clove lemon, horseradish, onion, garlic, rosemary, and oregano have been researched as antimicrobial agents [21,22]. In another study, a concept was proposed which says that bacterial infections, particularly those brought on by Gram-positive bacteria, can be prevented by lysozyme [23,24]. Its activity is explained by its capacity to hydrolyze the primary component of the cell wall, which results in the loss of intracellular components and bacterial death, which proves that enzymes can be used as an antimicrobial agent and can be incorporated in packaging.

## 3. Types of Antimicrobial Agent

### 3.1. Natural Antimicrobial Agent

There are several natural compounds which possess certain antibacterial activities. Some of these natural or organic compounds are used in the industry on a very large scale. One of the most significant sources of antimicrobial packaging is natural antibacterial agents. They are safe for health because they are natural. A crucial characteristic of antibacterial organic compounds is that they exhibit long life and high stability under specific conditions, such as heat, but they also have some drawbacks, such as weak mold-resistant activity; as a result, a large dosage is required when used professionally on an industrial scale. Some research has given a brief description of natural antimicrobial agents, the sources of some antimicrobial agents from animal and plant origins, the packaging materials in which they are frequently incorporated, the foods for which these packaging materials are also made, and the microorganisms active in these natural antimicrobial agents [25]. It also lists some antimicrobial agents’ sources from animal, plant, and microbial origins [26].

### 3.2. Plant-Based Antimicrobial Agent

The food sector is employing practical methods, such as the utilization of multiple plant-based compounds as a natural antibacterial agent in polymeric materials, to fully capitalize on the advantages and benefits of each material while also overcoming the downsides and shortcomings of each component. Individual volatile molecules are called essential oils (EOs). The effects of plant extracts employed as antibacterial agents in the form of thin edible films on food products are significant. These chemicals extended the shelf life of packaged foods and reduced waste [27]. They are obtained specifically from aromatic plants. Some of the most effective natural extracts are ginger, garlic, oregano, thyme, cinnamon, clove, coriander, and more [28]. The antimicrobial actions on particular microbes are brought on by the presence of active chemicals in these substances, such as flavanols, terpenes, anthocyanins, phenolic acids, tannins, and stilbenes. Additionally, they could offer other health advantages, similar to dietary supplements [29]. Ground beef’s shelf life was increased by up to 12 days using a composite made of PLA and nanocellulose infused with plant essential oils (*Mentha piperita* or *Bunium percicum*). Authors in [30] have created three-component composite films based on PLA blended with chitosan and packed with tea polyphenols in a range of molar ratios. The composite film made of PLA, tea polyphenols, and chitosan exhibits up to three times more strength. Thyme and tarragon are common plants that contain caffeic acid, which has a potent antibacterial, antiviral, and antifungal action. The phenolic structures of flavones only contain one carbonyl group. It should be noted that they are effective antibacterial compounds acting against a wide variety of microorganisms and are thought to be produced by plants in response to microbial infection.

In contrast to minor components like ketones and aldehydes, spice and herb extracts contain phenolic, terpene, and aliphatic alcohols with antimicrobial properties [31]. Besides the antimicrobial activity, they offer antioxidant activity and other medicinal effects [32]. From this perspective, using antimicrobial compounds originating from plant sources could be a great option, especially for food packaging. *Listeria*-infected cheese can be preserved for 24 days using starch films that have been infused with clove leaf oil. Clove leaf oil performs a variety of functions, including enhancing tensile strength and elongation break as well as reducing *L. monocytogenes* proliferation, acting as a UV barrier, and scavenging free radicals [33]. The loss of plant-derived antimicrobial compounds during high-temperature processing and decreased antibacterial effectiveness are the current problems with plant-derived antimicrobial compounds [34]. Plants that contain phytochemicals are generally essential, especially those that have therapeutic advantages [35,36,37,38,39]. Due to rising consumer demand for safer food additives, numerous studies and evaluations have been carried out in photochemistry about natural antimicrobial agents. Table 1 describes the sources of some antimicrobial agents from plant origins.

### 3.3. Animal and Microbe-Based Antimicrobial Agent

Mostly natural agents derived from plants were discussed in the previous section. In this section, antimicrobial agents derived from animal and microorganisms are briefly discussed. Today, a variety of strategies are employed to increase the effectiveness and production of natural antibacterial agents. These methods have enhanced the effectiveness of natural antibacterial agents and ensured the safety of food packaging. Pathogens are currently a big hazard to the food business. Dealing with the condition is highly challenging because the infections have rapid growth rates and develop a resistance to the antibacterial agents. In this situation, researchers put forth a lot of effort to overcome this problem. Hossain et al. 2017, in their study, explained that probiotics are the live microbes that are provided to humans and animals [50]. They act as the antibacterial agents in the intestinal tract and can fight harmful microbes in the ingested food. Bacteriocins from microorganisms (natamycin and nisin), as well as lactoferrin and lysozyme enzyme from animals, are natural preservatives used in food packaging [51]. In the twenty-first century, animal-derived antimicrobial agents are frequently employed in food packaging. The hypothetical study leads to the conclusion that sea cucumber is very significant from a medical and dietary standpoint. It is believed to have been used in the past to treat wounds, and current research suggests that it possesses antibacterial and antioxidant qualities. In most of Asia’s regions today, it is employed in the food and pharmaceutical industries [52]. Next to cellulose, lignin is the second-most prevalent renewable and biodegradable natural resource. It includes a variety of functional groups in varying proportions, giving room for chemical modification and polarity adjustment to produce compatibility with suitable polymeric matrices [53]. The literature has determined that a variety of polymeric substances derived from animals, such as chitosan, protein hydrolysates, bioactive peptides, whey protein, etc., display innate antibacterial activity. Various bacteria, including *Escherichia coli*, *Bacillus cereus*, *Salmonella typhimurium*, *Staphylococcus aureus* and *Listeria monocytogenes*, as well as mold and yeast, including *Rhizoctonia solani*, *Botrytis cinerea*, *Fusarium oxysporum*, and *Candida lambica*, have shown antagonistic behavior toward chitosan [54]. Natural antimicrobials, such as bacteriocins, have a “green” nature and are well-known for their antimicrobial efficacies. In order to prevent synthetic antimicrobials from migrating into food, great effort has been paid to substituting naturally occurring antimicrobials with those found in synthetic ones. Bacteriocins are ribosomal-generated antimicrobial peptides made by bacteria that can prevent or eradicate other closely related bacteria from multiplying [55]. The various types of sustainable antimicrobial materials derived from animal and microorganism sources are updated, and future trends are examined, along with their compositions, traits, antimicrobial mechanisms, and food applications. Despite their impressive properties, more research is needed to confirm the materials’ safety and effectiveness.

### 3.4. Chemical Antimicrobial Agent

As an antibacterial, organic acids and their salts are used in food packaging. Sorbic, propionic, lactic, acetic, and benzoic acids make up the majority of organic acids. By transferring nutrients and metabolizing them, they compromise the integrity of microorganisms’ cell membranes and macromolecules [56]. Inorganic, organic, and biologically active compounds are among the antimicrobial agents employed in the creation of antimicrobial packaging materials [57]. The implementation of organic acids as antimicrobial agents in the food material depends on several characteristic properties of the acids, such as the chemical formula, physical form, pKa value, molecular weight, minimum inhibitory concentration, nature of the microorganism, buffering properties of the food, and acid–food exposure time [58]. These tests have shown that it is possible to make antimicrobial packaging from substances that are already used in the food business, typically in the form of a nanocomposite film. One of the biggest issues with the adoption of new technologies is the rise in production costs. With only minor changes to the production lines, the industry will adopt new technologies that can be reverse-engineered from the ones already in use. These adjustments may result from new rules, projections of higher earnings, shifts in public opinion and demand, or other factors [59]. The second most popular polysaccharide utilized to make edible films and biodegradable packaging is cellulose. It is available from a wide range of bacterial sources. Acidic hydrolysis can produce cellulose nanocrystals from bacterial cellulose (BC) from *Gluconacetobacter xylinus*. The factors limiting the applicability domains include the low mechanical performance and the lack of water resistance. The antimicrobial agent is applied to the cellulose to boost the added value of the packaging materials [60]. Organic acids, which are widely used in the industry for diverse purposes [61], can be generated by numerous microorganisms; the biological manufacture of organic acids via microbial fermentations has advantages over the chemical production techniques, such as cost-effectiveness, practicality, reliability, environmental friendliness, sustainability, and reduced carbon footprints (Table 2). 

## 4. Potential Applications of Antimicrobial Packaging in Food

**Figure 1:**
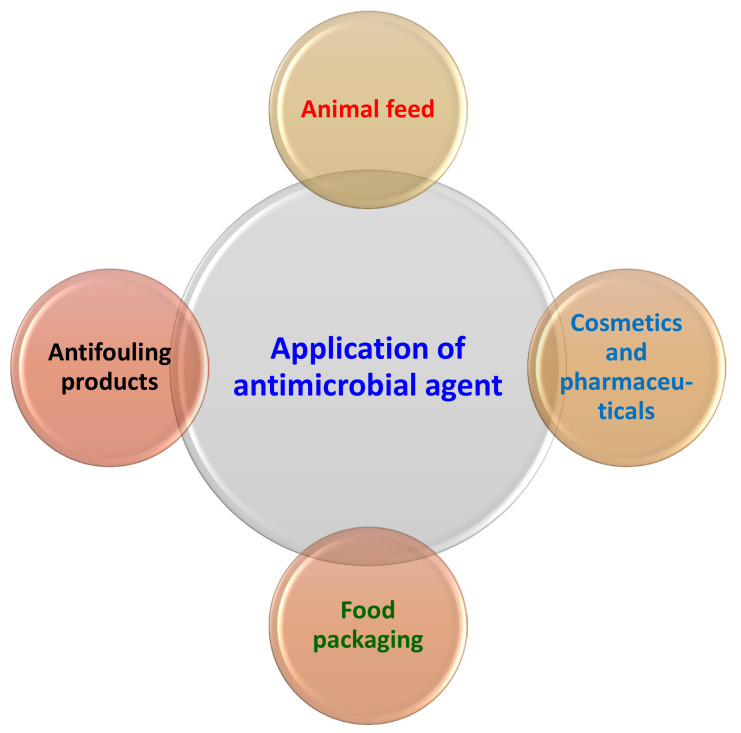
Various applications of antimicrobial agent.

Antimicrobial agents are useful for many applications, including packaging and the pharmaceutical industry (Figure 1). Recently, there has been a huge growth in the need for biodegradable and renewable materials for packaging applications. There is a market demand for healthy and natural food products, and the strict guidelines to prevent infectious diseases that are spread by food; recalls have inspired researchers to develop new methods of delivering antimicrobials, which should lead to enhancing the food products’ quality and safety over the course of storage. A promising type of active food packaging that is still in its infancy is antimicrobial packaging. Regulations for food packaging require a fresh strategy. Although promising, antimicrobial food packaging currently only has a few applications. This is as a result of the tested additives’ status as authorized substances [71]. Furthermore, microbial activity is a major concern in the food packaging sector. Therefore, using antimicrobial agents or polymers to create barrier-enhanced or active packaging materials offers a desirable option for guarding against the growth and spread of microorganisms on food [72]. The use of an antimicrobial agent should be combined with biodegradable packaging materials for a more thorough approach. This is due to the fact that the use of biodegradable packaging films is currently a highly recognized global trend, particularly when the biodegradable components are generated from renewable resources. The application of antimicrobial agents in different food systems is briefly represented in Table 3.

Antimicrobial packaging is crucial for food packing as it may increase the shelf life and ensure food preservation. In terms of food product and food packaging films, antimicrobial packaging systems have offered various innovative technologies, among which one of the trending and most focused systems is the concept of active packaging. One of the most innovative active packaging technologies is antimicrobial packaging, which has several uses, including oxygen-scavenging packaging and moisture-controlling packaging [79]. In this sector, there is growing interest in edible packaging films due to environmental concerns of synthetic polymer-based packaging materials. The concept of biodegradable active packaging has been improved with edible packaging films with antibacterial coatings. On the surface of food goods, they have been employed to lessen and prevent the growth of germs through chemical and physical mechanisms. An antimicrobial packaging (AM) system seeks to safeguard packaged foods against various microbes [80]. 

Nanocrystalline cellulose (CNC) and cellulose nanofibers (CNFs) are biopolymers that are abundantly available in nature, biodegradable, non-toxic, and renewable. They can be extracted from lignocellulosic plants or produced by microbes. Nanocellulose (NC), which has a high surface-to-volume ratio and special physicochemical characteristics such as surface chemistry, high crystallinity, mechanical strength, and morphology in nanometer structures, enhances the packaging properties of nanocomposites such as coatings and nanofillers in films (81). On the other hand, the industry could also benefit from the creation of gelatin and other biodegradable films by using less water, solid waste, electricity, and emissions. Additionally, biopolymer-based films have a strong matrix and compatibility that makes it possible for antimicrobial and antioxidant agents to be incorporated into the film and perform their respective tasks for extending the shelf life, functionality, and safety of food products (82). Research was conducted earlier (83) to create smoke-flavored antimicrobial packaging using coconut fiber manufactured from *Litsea cubeba* oil at 0.03%, 0.06%, and 0.09% w/w mixed with wood smoking for 30, 45, and 60 min. The goal was to extend the shelf life of dried fish and check the antibacterial effect and smoky flavor. *Aspergillus niger* was completely prevented from sprouting on the outermost layer of dried fish by the prepared packaging material. Additionally, the treated packaging produced volatile compounds to keep dried fish fresh for at least 21 days at room temperature (30 °C) without the growth of bacteria or mold.

Future studies are likely to combine biopreservatives, biodegradable packaging materials, and naturally produced antimicrobial agents, which will highlight a wide variety of antimicrobial packaging in regards to food safety, longevity, and environmental friendliness. A helpful technology in antimicrobial packaging systems successfully charges the antimicrobial properties within the food packaging film medium and eventually produces it over the necessary period of time to kill the harmful microbes influencing food products [81]. The use of natural antibacterial chemicals is one of the new technologies that has gained popularity because it has no hazardous or unfavourable impacts on consumers. A study [82] has proven that the allyl isothiocyanate (AITC) from ground mustard seeds, when incorporated in designing active antimicrobial packaging, possesses the ability to inhibit the growth of microorganisms. PHB, PLGA, and starch derivatives all have properties that make them suitable for various antimicrobial packaging agents. In biomedical and food applications, they have been examined for both harmful and spoilage microorganisms under various testing settings [83]. The perfect AM polymer films should therefore satisfy a number of key requirements, with the ease and affordability of the AM film production process being the most crucial in the food business. Second, for long-term use and storage, the films must be chemically stable. As a water barrier and to preserve the AM effect during the packing period, the AM film must also be durable. It is crucial that the packaging materials for AM do not release dangerous particles into food and are safe to handle. However, due to factors including inadequate understanding regarding the effectiveness of AM in polymer films, economic impact, consumer acceptance, and a lack of particular rules surrounding active packaging, the development and deployment of natural AM active packaging is constrained.

**Figure 2:**
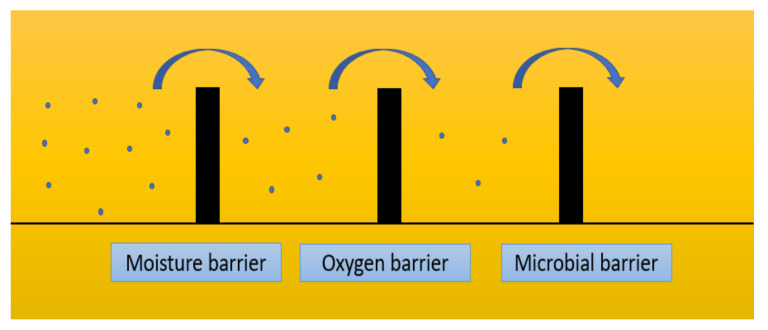
Hurdle technology in antimicrobial packaging system.

The antimicrobial packaging system is a hurdle to preventing degradation of total quality of packaged food, providing protection against microorganisms. The application of preservative hurdles like low storage temperature, the addition of antimicrobials and/or antioxidants, water activity, pH, and high-pressure processing with alternative packaging, such as modified atmospheres, has generally shown the potential to further extend the shelf life of food products, as diagrammatically represented in the above Figure 2. The Figure 2 describes the hurdle technology in terms of moisture barrier, oxygen barrier and antimicrobial barrier to improve the life span of packed food. Contrary to popular belief, biodegradable packaging can be created from both bio-based and plant-based materials. The strength and molecular makeup of a material’s polymer chain determine its capacity to degrade, not its origin [84]. In addition to serving as packaging, biodegradable packaging has a number of uses in the food industry, including extending the shelf life of packaged foods and slowing the growth of microorganisms that come into direct contact with food. In addition to being antibacterial, the packaging is also sanitizing and self-sterilizing. In order to increase shelf life and guarantee the microbial safety of fresh and minimally processed produce during storage, a number of antimicrobial packaging systems have been created. These systems deliver a continuous and controlled release of the active antimicrobial substances into the package [85]. A recent study created antibacterial and antiviral films, where silver ions were successfully integrated into polylactide acid (PLA) films [86]. These films have been utilized to package lettuce samples contaminated with Salmonella enterica. Six days of storage at 4 °C resulted in a 4 logCFU reduction in S. enterica in packaged lettuce. A polymer matrix capable of spreading antimicrobial chemicals uniformly is necessary for the production of antimicrobial packaging films. Although the scientific literature frequently reports on the antibacterial and antioxidant properties of nanomaterials and plant extracts in different polymer combinations [87], in order to do this, it has become more advantageous in recent years to produce antimicrobial films using biodegradable material as opposed to non-degradable material. 

## 5. Nanotechnological Interventions of Green Antimicrobial Packaging

**Figure 3:**
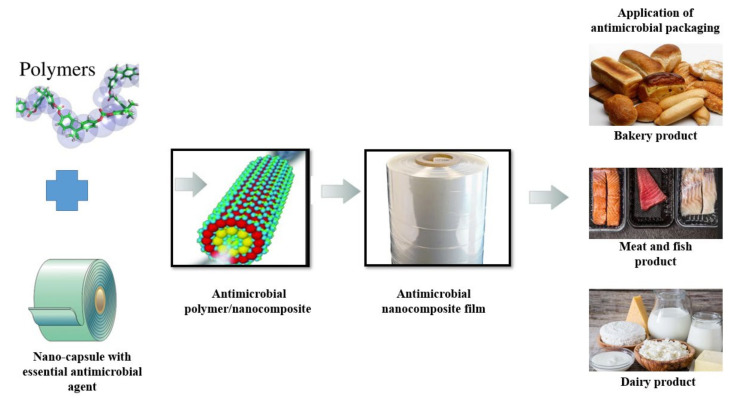
Preparation of nanostructured film incorporated with antimicrobial agent.

The purpose of packaging is to maintain the quality and texture, protect from contamination by germs, chemicals, and physical agents, and create a consumer-friendly presentation. Additionally, current approaches to environmentally friendly NP synthesis, based on the use of plant extracts and biomolecules as reducing and complexing agents in nanocomposite films, have demonstrated significant potential for palatable, biodegradable, and sustainable packaging [88]. A paradigm change from various chemical-based technologies to a greener and more sustainable approach has been brought about by growing environmental concerns. Recent discussions have focused on the development and potential of multifunctional nanocomposites based on antimicrobial agents in both the academic and industrial sectors. Nanotechnologies are rising very fast and gaining great popularity to support the benefits of the preservation of foods [89]. Nanoparticle-infused inorganic and metal oxides are also being explored. Due to their high stability and effective antibacterial properties, metal oxide-based nanoparticles such as ZnO, MgO, CuO, and TiO_2_ have been investigated for use as antimicrobial agents in food packaging [90]. Recent green nanotechnological interventions combine biological sources, cleaner solvents, recyclable materials, and energy-saving procedures to create nanoparticles for use in food processing, packaging, and preservation (Figure 3). Many studies have already been conducted on various nanofillers derived from diverse sources, and more studies are being conducted to identify nanofiller-reinforced bionanocomposites that can be successfully applied for food packaging applications [91]. Additionally, a number of cutting-edge nanoencapsulation technologies have been created that use a number of biocompatible delivery systems as a carrier for a number of bioactive and nutritious components to enable regulated release and improved stability for food processing and preservation [92].

**Figure 4:**
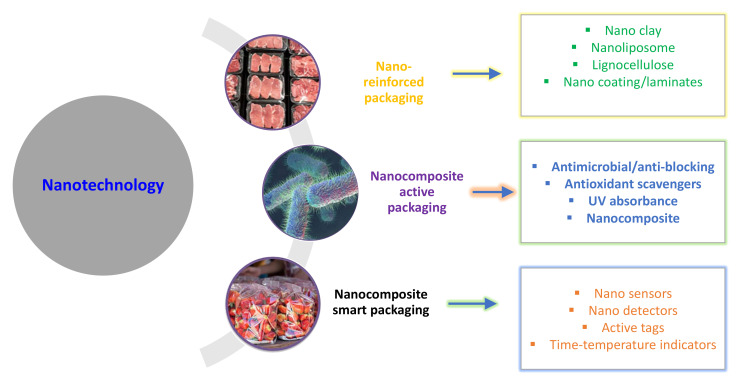
Application of nanotechnology in food packaging industry.

In the food packaging industry, new antimicrobial, antioxidant, and sustainable systems have been vigorously promoted as a viable eco-friendly alternative to conventional materials for improving the quality and safety of food products while minimizing or eliminating their negative environmental effects (Figure 4). The encouragement of more effective assembly and subsequent release of environmentally friendly active principles is made possible by the use of nanotechnologies, which also limits the usage of chemicals in terms of associated financial losses. Nanotechnology has the potential to completely alter the situation and satisfy the growing demand for global sustainability. Nevertheless, applying sustainable management measures that also take a nanotechnological approach to the full agri-food chain, from the plant to the food items, may be of interest.

The usage of nanoparticles in food packaging, on the other hand, is anticipated to grow to USD 20 billion by 2020, according to the European Institute for Health and Consumer Protection [93]. The protection of the antimicrobial packaging against early inactivation in the food matrix and the regulated release of the drug, allowing a potential extension of shelf life, would be some benefits of nanotechnology [94]. As a result, nanotechnology has great promise for enhancing food safety and serving as an effective vehicle for the delivery and regulated release of natural antimicrobials [95,96]. Recently, the potential presented by a multifunctional system approach including sustainable supplies and greener methodologies was studied in connection to poly (lactic acid) (PLA)-based composites made using microcrystalline cellulose mixed with silver nanoparticles. Various stabilizing issues can be effectively solved by incorporating nanosized metals into biodegradable polymer matrices, which also allows for a controlled antibacterial effect [97]. The ability to make use of nanotechnology is considered compatible with the large-scale; roll-to-roll manufacturing of nanocomposite PLA films, as required by packaging technology, is provided by the simplicity of nanoparticle creation and the fact that they are obtained as a dispersion [98]. In the 1990s, research on the application of innovative packaging solutions, including those utilizing nanomaterials, nanocomposites, etc. [99], conducted a biological experiment against *Pseudomonas* spp. in which it was concluded that the suggested nanocomposites have strong antibacterial activity, making them a desirable nanomaterial.

## 6. Sustainable Contribution to the Society

The imminent threat that climate change poses to our society is constantly discussed in the media, but humanity’s current use of resources is still not in line with the goal of sustainable development. In order to overcome the problems, changing how food is consumed is essential. A significant portion of the impact is attributable to agriculture and food production, and this is also true for food consumption. The enormous breakthrough that is globalization and the subsequent economic development are among the results of society’s ongoing evolution and search for new solutions. Environmental considerations are now being given more weight in package development trends worldwide. Unfortunately, after being used, the packaging becomes waste, which can have a negative impact on the environment, which is why these concepts are also increasingly taken into account by companies from the packaging industry. In order to prevent the loss of goods, the packaging industry is intended to be transformed into one that is environmentally and socially conscious through the introduction of certain legislative requirements. According to SPA (an American organization, the Sustainable Packaging Coalition), packaging should be safe, effective, efficient, and cyclical [100]. Manufacturers are searching for novel solutions by developing creative biodegradable packaging materials or employing renewable raw materials in order to adapt the industry to the current requirements and changes. However, improving certain areas related to production, use, or disposal can sometimes be time- and labor-intensive, but not impossible. 

Plastics are one of the most commonly utilized materials in the creation of packaging on a global scale, largely because of their adaptability and versatility. Contrary to popular belief, they are particularly well-suited for the creation of sustainable packaging since they offer protection, are long-lasting, and because of the package’s light weight, they lower transportation costs and related greenhouse gas emissions. Additionally, they are easily moldable into any shape [101]. Unfortunately, they are often not managed properly after use and this is a global problem. Paper goods are utilized extensively in the creation of packaging all around the world in addition to plastics. They produce unit packaging, wrappings, labels, and, most importantly, transport packaging. Their biodegradability is their main selling point. Unfortunately, this process results in the production of methane, a greenhouse gas [102]. 

**Figure 5:**
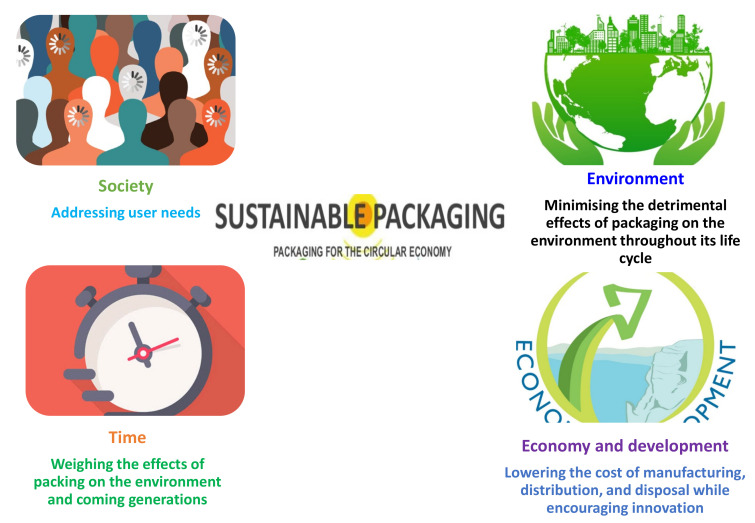
Sustainable packaging solution for the circular economy.

Sustainability is a multifaceted concept. In packaging, there are five fundamental categories as the cornerstones of sustainable packaging, i.e., society, environment, economy, development, and time, which are outlined in Figure 5 [103]. Due to the antimicrobials’ absorption into the polymeric matrix, the previous non-compostable oil-derived polymers were replaced with antimicrobial biodegradable packaging materials, which presented additional issues. By improvising this, we can enhance the packaging system present on the market, but they should also be constantly improved and adapted to the changing requirements of the market, society, or the environment, as well as to the state and development of the packaging industry. More and more businesses are making efforts to transform the packaging sector into one that is socially and environmentally conscious. They take into account packaging from an economic, social, ethical, and environmental perspective. The answer for the various problems lies in rebuilding our industrial systems so that they are intrinsically sustainable rather than trying to mitigate their negative elements. More sustainable packaging will be a key component of such future systems.

## 7. Conclusions and Future Prospects

Efficiency and innovation in packaging technologies have become a key component of the new regulations used to ensure food preservation and protection due to the constantly increasing demand for minimally processed food products and the resulting expansion of the market for those products. Due to the addition of the antimicrobial compounds into the polymeric matrix, compatibility between diverse components, and ease of degradation by heat and light, the antimicrobial biodegradable packaging materials that replaced conventional non-compostable oil-derived plastics presented new hurdles. The use of antimicrobial components in the packaging such as natural bioactive compounds, peptides, nanoparticles, etc., in food packaging helps to prevent undesirable changes in the food during storage, such as off flavors, color changes, or the occurrence of any foodborne outcomes. It also ensures controlled release over an extended period to improve the quality of food under observation and, overall, improves the quality in terms of value for money for the customers. While all of these antimicrobial packaging systems are somewhat successful at maintaining fresh and minimally processed produce’s quality and ensuring its microbial safety, more research and development are required to increase the antimicrobial effectiveness of the current packaging technologies, find more potent natural antimicrobial compounds, increase the stability of natural antimicrobials in the packaging system, and guarantee the safety of their commercial application. When using nanoparticles in food products, thorough toxicity studies, safety precautions, and exploration of nano-based antimicrobial packaging techniques must be undertaken. The fundamental benefit of adding antimicrobial components to food packaging materials is that they gradually release into the food surface, allowing for continuous antibacterial activity and extending the shelf life of the food. However, more research on the in vitro and in vivo performances of this active packaging material is required in order to define regulation in this context. The development of techniques and technologies aimed at preserving food products and enhancing food safety and quality can still take advantage of this promising field. Future research is needed to increase the longevity and effectiveness of novel antimicrobial packaging materials because some antimicrobials (such as essential oils) have a high loss rate due to inherent volatilization. The limits of what is practical for sustainable packaging will continue to be pushed by advancements in design, production, and recycling technology. Researchers are working arduously to contribute to the most advanced solution for this issue.

## Figures and Tables

**Table 1:** Active compound isolated/extracted from natural antimicrobial agents.

Natural Extract	Active Compounds	Antimicrobial Action	Reference
Oregano	Carvacrol and thymol	*Salmonella* enteric, mold and yeast, and mesophilic aerobic bacteria	[40]
Clove and thyme	Eugenol and thymol	*Escherichia coli*	[41]
Eucalyptus radiata	eucalyptol	Gram-negative bacteria	[42]
Grapefruit seed	phenolic compounds	*Pseudomonas fluorescence* and *Escherichia coli*	[43]
Apricot kernels	oleic acid	*Escherichia coli* and *Bacillus subtilis*	[44]
cinnamon	cinnamaldehyde	*R. nigricans* and *S. aureus*	[45]
Basil	Chavicol and eugenol	*L. curvatus* and *S. cerevisiae*	[46]
Tea polyphenols	Polyphenols	*Escherichia coli* and *Staphylococcus aureus*	[47]
*Mentha rotundifolia* L.	Ferulic acid	*Salmonella typhimurium*, *Escherichia coli*, *S. aureus*	[48]
Olive leaf extract	polyphenolic compounds	*Escherichia coli* and *L. innocua*	[49]
Chitosin	polycationic compounds	*S. aureus*, *Listeria monocytogenes*, and *Enterococcus faecalis*	[50]
Bacteriocins	Peptide	*Micrococcus luteus*, *S. aureus*, and *Bacillus cereus*	[51]
Cellulose		*Staphylococcus aureus*, *Escherichia coli*, and *Candida albicans*.	[52]
Starch		*Escherichia coli*	[53]
Lysozyme		Gram-positive bacteria	[54]

**Table 2:** Commonly used organic acids in the food industry.

Organic Acids	Antimicrobial Action	Reference
Malic acid	*L. monocytogenes*, *S. gaminara*	[62]
Phenyllactic acid	*E. cloacae*	[63]
Propionic acid	*E. coli* and *Salmonella*	[64]
Citric acid	*Sh. flexneri*	[65]
Sorbic acid	*Yeast* and *mold*	[66]
Oxalic acid	*Escherichia coli*	[67]
Acetic acid	*Shigella*	[68]
potassium sorbate	Bacteria and molds	[69]
Sodium citrate	*Listeria*, *bladder*, and *Escherichia coli*	[70]
Allyl isothiocyanate	*E. coli*	[71]

**Table 3:** Antimicrobial agents’ antimicrobial efficacy in various dietary products.

Antimicrobial Agent	Food Product	Targeted Microorganism	Reference
triclosan	Meat	*Triclosan*, *S. aureus*, *B. thermosphacta*	[73]
wasabi extract AM	Raw meat	*E. coli* and *S. aureus*, fungi *A. niger*, *P. italicum.*	[74]
Olive leaf extract	cheese	*S. aureus*	[75]
Grape seed extract	pork loin	*L. monocytogenes*, *E. coli*, *E. faecalis*, *E. faecium*, *S. typhimurium*, and *B. thermosphaceta B2*	[76]
Chitosan and cinnamaldehyde	vacuum-packed cured meat products (bologna, cooked ham, and pastrami)	*Enterobacteriaceae*, *Serratia liquefaciens*, and *Lactobacillus sakei*	[77]
Corn zein	Ready-to-eat chicken	*L. monocytogenes*	[78]
Pimento EOs	Beef muscle slices	*Pseudomonas* spp. and *E. coli* O157:H7	[79]

## Data Availability

Not applicable.
